# Breaking Barriers to an AIDS Model with Macaque-Tropic HIV-1 Derivatives

**DOI:** 10.3390/biology1020134

**Published:** 2012-07-05

**Authors:** Rajesh Thippeshappa, Hongmei Ruan, Jason T. Kimata

**Affiliations:** Department of Molecular Virology and Microbiology, Baylor College of Medicine, Houston, TX 77030, USA; Email: thippesh@bcm.edu (R.T.); hruan@bcm.edu (H.R.)

**Keywords:** HIV-1, SIV, *Macaca nemestrina*, AIDS, cross-species, tropism, innate restriction

## Abstract

The development of an animal model of human immunodeficiency virus type 1 (HIV-1)/AIDS that is suitable for preclinical testing of antiretroviral therapy, vaccines, curative strategies, and studies of pathogenesis has been hampered by the human-specific tropism of HIV-1. Although simian immunodeficiency virus (SIV) or HIV-1/SIV chimeric viruses (SHIVs)-rhesus macaque models are excellent surrogates for AIDS research, the genetic differences between SIV or SHIV and HIV-1 limit their utility as model systems. The identification of innate retroviral restriction factors has increased our understanding about blockades to HIV-1 replication in macaques and provided a guide for the construction of macaque-tropic HIV-1 clones. However, while these viruses replicate in macaque cells *in vitro*, they are easily controlled and have not caused AIDS in host animals, indicating that we may not fully understand the restrictive barriers of innate immunity. In this review, we discuss recent findings regarding HIV-1 restriction factors, particularly as they apply to cross-species transmission of primate lentiviruses and the development of a macaque model of HIV-1/AIDS.

## 1. Introduction

A significant problem for testing novel antiretroviral drugs and vaccines against human immunodeficiency virus type 1 (HIV-1) has been the lack of an animal model due to the inability of the virus to replicate in species other than humans. Although chimpanzees and gibbon apes can be experimentally infected with HIV-1, AIDS-like disease is not typically observed. A recent report suggests that simian immunodeficiency virus cpz (SIVcpz), the precursor of HIV-1, may cause AIDS in wild chimpanzees [1]. However, it is not clear how commonly this happens. Moreover, the endangered status and the high maintenance cost associated with apes make them unsuitable model hosts for experimental studies of HIV-1 [2]. Other nonhuman primate species, including Asian macaques that are used for the SIVmac AIDS model, have not proven to be susceptible to HIV-1. Thus, this narrow host range of HIV-1 has complicated the development of a nonhuman primate model of HIV-1 infection and disease. 

In the past several years, studies have identified anti-retroviral factors, collectively called intrinsic immunity, in human and nonhuman primate cells that restrict infection. Accessory proteins expressed by HIV-1 (*i.e.*, viral infectivity factor (Vif), viral protein U (Vpu), viral protein R (Vpr) and negative factor (Nef)) have been shown to play key roles in overcoming intrinsic immunity in human cells. Thus, successful transmission of HIV-1 to nonhuman primates may depend on whether these accessory proteins function in model host species. Here, we review the literature about innate barriers to HIV-1 infection and application of this knowledge to developing a macaque model of HIV-1/AIDS. 

## 2. Cross-Species Transmission of HIV-1

The emergence of HIV-1 is believed to have started with the transmission of SIVcpz from chimpanzees (*Pan troglodytes*) to humans [3]. SIVcpz entered the human population on at least three independent occasions, with each transmission event giving rise to HIV-1 groups M, N, and O [4]. Recently, a new HIV-1 that is closely related to an SIV that infects gorillas (*Gorilla gorilla gorilla*) (SIVgor) has also been identified. This virus appears to represents a fourth transmission lineage (HIV-1 group P) [5]. Interestingly, SIVcpz is believed to have evolved from successful infection and recombination of two SIV strains, SIVgsn from greater spot-nosed monkeys (*Cercopithecus nictitans*) and SIVrcm from red capped mangabeys (*Cercocebus torquatus*), in chimpanzees [6]. During recombination, SIVcpz acquired accessory genes such as *vpu* from SIVgsn and *nef* from SIVrcm. A recent report suggests that host dependent adaptation of *vpu* and *nef* may have enhanced replication of SIVcpz in chimpanzees [7]. Other recent studies provide additional insights on adaptive mutations in SIVcpz genes that may have enabled it to jump from chimpanzees to humans [7,8,9,10]. Thus, these evolutionary studies on the origin of HIV-1 suggest that primate lentiviruses may be highly dependent on the functional activity of accessory genes for jumping into new species. 

## 3. SIV and SHIV Macaque Models

Nonhuman primate models have been developed to study AIDS pathogenesis and to evaluate the efficacy of vaccines and drugs. The earliest animal model developed was infection of rhesus macaques (RM, *Macaca mulatta*) with a simian immunodeficiency virus. Many African monkey species harbor SIVs and live with high virus loads without developing disease [11,12,13,14,15,16,17]. However, SIV isolated from sooty mangabeys (SM, *Cercocebus atys*) can cause AIDS when inoculated into RMs [18,19]. Infectious molecular clones of SIVsm, named SIVmac, were generated from the infected RMs and shown to cause AIDS in naïve RMs [20,21]. SIVmac has also been shown to cause AIDS-like disease in cynomolgus monkeys (CM, *Macaca fascicularis*) and pig-tailed macaques (PTM, *Macaca nemestrina*) [22,23]. Consequently, SIV infection of macaques is widely used as a model for AIDS pathogenesis. This SIV model has also helped provide an understanding of the importance of viral genes, such as *vpx*, *vpr* and *nef* in SIV replication *in vivo* [24,25,26,27,28,29,30]. 

Although the SIV-macaque model has been informative for studies of pathogenesis, it does have some shortcomings (reviewed in Ambrose *et al*. [31]). A primary concern is the genetic differences between HIV-1 and SIVmac. For example, HIV-1 encodes *vpu*, which makes it difficult to study its role in pathogenicity since a homolog does not exist in SIVmac. Additionally, the *vpx* gene is present in SIVmac but not HIV-1. There are also differences in the reverse transcriptase (RT) and protease (Pr) enzymes [32,33], which make it difficult to evaluate the efficacy of drugs targeting these enzymes when using SIVmac as the model virus. For example, non-nucleoside RT inhibitors block the HIV-1 RT but not SIV RT [34]. Finally, the differences in sequences of HIV-1 and SIV may affect humoral and cellular immune responses. First, the viruses are not likely to share immunodominant cytotoxic T-cell (CTL) epitopes. Second, structural differences in the HIV-1 and SIV Env proteins may lead to qualitative differences in the antibody responses [35,36]. Moreover, neutralizing antibodies to the SIV Env are not crossreactive with the HIV-1 Env [37]. Additionally, there could be qualitative differences in the immune response to HIV-1 compared to SIV in nonhuman primates. Together, these differences limit the utility of the SIV-macaque model for translational studies of antiviral therapy or vaccines. 

To overcome some of the limitations of the SIV-macaque model for vaccine and antiretroviral inhibitor studies, chimeric viruses that incorporate parts of the HIV-1 genome into the background of SIV have been generated. Shibata *et al*. constructed initial SIV and HIV-1 chimeric viruses (SHIVs) by inserting HIV-1 genes such as *tat*, *rev*, *vpu* and *env* into SIVmac239 backbone [36]. Subsequently, several independent groups showed that novel SHIVs could replicate and cause disease after passing the viruses through macaques [38,39,40]. This model has helped increase our understanding of the role of envelope in pathogenesis [41,42,43]. It has also been useful for determining the efficacy of passively transferred neutralizing antibodies [44,45,46,47], vaccines [48,49], and microbicides [50] in preventing SHIV infection. To study the efficacy of non-nucleoside inhibitors of reverse transcriptase (RT), chimeric viruses in which the RT of SIV has been replaced with that from HIV-1 (RT-SHIV) have also been developed [34,51,52,53,54,55,56]. While these SHIVs have improved utility, the remaining issue is that these viruses mainly consist of SIV sequences and single HIV-1 genes. Thus, it is not possible to test vaccines or antiretroviral drugs targeting multiple HIV-1 proteins. On the other hand, while there are shortcomings to the SHIV-macaque model for translational studies, the construction of pathogenic SHIV chimeras revealed which HIV-1 genes are functional in macaque hosts, narrowing the determinants that may be critical for cross-species transmission of HIV-1 into macaques. 

## 4. Innate Cellular Restriction Factors

### 4.1. Overview

In the last decade, cellular factors have been identified that inhibit retroviral replication at different stages of the life cycle. Although HIV-1 and SIV can overcome these restriction factors in their natural hosts, they may not do so in other species, suggesting that innate restriction factors act as barriers for cross-species transmission of HIV-1 and SIV. The four major restriction factors that have been identified are the apolipoprotein B mRNA editing enzyme catalytic polypeptide 3 (APOBEC3 or A3) family of proteins, tripartite motif containing (TRIM) family of proteins, BST2/CD317/Tetherin, and sterile alpha motif (SAM) and histidine/aspartic acid (HD) domain containing protein 1 (SAMHD1) (Table 1). Interestingly, each is upregulated by type 1 interferons, linking restriction to the innate immune response. Additionally, accumulating evidence indicates that each gene has been under strong positive selection in nonhuman primates and humans in response to lentiviral infections, further indicating their importance for controlling infection [57,58,59,60,61,62,63]. 

### 4.2. APOBEC3 Proteins

The APOBEC3 family members are cytidine deaminases. This family is comprised of seven members in humans: A3A, A3B, A3C, A3CD, A3F, A3G, and A3H [64]. The antiviral activity of APOBEC3 family proteins was first identified by Sheehy *et al*. , who discovered A3G as a factor that renders Vif deficient HIV-1 progeny virions non-infectious in non-permissive cells but not permissive cells [65]. In the absence of Vif, A3G is packaged into HIV-1 progeny virions [66]. This requires an interaction of A3G with the nucleocapsid region of the Gag protein and viral or cellular RNAs (reviewed in [67]). Only a few incorporated A3G molecules are enough to exhibit antiviral activity [68]. When the progeny virions infect the next target cell, the incorporated A3G deaminates cytidine (C) to uracil (U) in the minus strand DNA during reverse transcription. Uracil then codes for adenine during the synthesis of positive strand DNA, resulting in accumulation of deleterious G to A mutations in the viral DNA [69,70,71,72]. G to A mutated genomes either undergo degradation [73] or lead to expression and degradation of truncated or misfolded proteins. Processed peptides may enhance recognition of infected cells by CD8^+^ cytotoxic T lymphocytes [74]. Additional family members of APOBEC3, such as A3F, A3C, and A3D have also been shown to affect the replication of HIV-1 [75,76,77,78,79]. Finally, A3G has also been shown to affect HIV-1 replication in a cytidine deaminase independent mechanism by inhibiting reverse transcription of viral RNA [80,81,82]. 

Vif inhibits the antiviral activity of APOBEC3 family proteins by preventing their incorporation into progeny virions. Several reports have shown that Vif interacts with A3G and facilitates its degradation by directing it to the proteasome [66,83,84,85,86,87]. Proteosome mediated degradation of A3G requires an interaction of Vif with ElonginB-ElonginC-Cullin5 E3 ligase complex. This Vif-E3 ligase complex then ubiquitinates A3G, which undergoes proteasome mediated degradation. Some reports indicate that Vif may also downregulate A3G expression [88,89]. 

**Table 1:** Restriction factors and the counter measures.

Restriction Factors	Mechanism of Inhibition	Counter Measures Used by Lentiviruses	Species–Specific Counter Measures		
			Restriction Factors	Inhibited by:	Resistant to:
ABOBEC3 family	Introduce G to A mutations, reduce infectivity, may affect reverse transcription	Vif prevents encapsidation of APOBEC3 family proteins in to the progeny virions	RM, AGM A3G	SIVmacVif, SIVagmVif	HIV-1 Vif
			CPZ, hA3G	HIV-1 Vif	SIVagmVif
BST2	Restrict release of mature progeny virions from the cell surface	HIV-1 Vpu, SIV Nef, and HIV-2 Env overcome BST2 function either by degrading or downmodulation of cell surface expression	CPZ, hBST2	HIV-1 Vpu	SIVmacNef
			RM, PTM BST2	SIVmacNef	HIV-1 Vpu
SAMHD1	Reduces dNTP pool required for cDNA synthesis	Vpx leads to proteasome mediated degradation of SAMHD1	Human, gibbon, RM SAMHD1	Vpx from HIV-2rod, SIVmac, and SIVsm	HIV-1 Vpr
**Restriction Factors**	**Mechanism of Action**	**Escape Mechanism**	**TRIM Proteins**	**Resistant Viruses**	**Inhibited Viruses**
TRIM family	Bind to incoming viral capsid and block infection at or before reverse transcription	Viral capsid mutations confer resistance to TRIM5 family proteins in their natural host	RM, CM TRIM5α	SIVmac	HIV-1
			Human TRIM5α	HIV-1	N-MLV, SIVmac
			AGM TRIM5α	SIVagm	HIV-1, HIV-2, SIVmac
			OWM TRIMcyp	SIVmac	HIV-1
			RM TRIMcyp	HIV-1	HIV-2, SIVmac
			PTM TRIMcyp	HIV-1	FIV, SIVagm, HIV-2

Several studies have shown that Vif counteraction of A3G occurs in a species-specific manner [90,91,92,93,94]. For example, RM and African green monkey (AGM, *Chlorocebus aethiops*) A3G are not recognized by the HIV-1 Vif protein and therefore block HIV-1 infection; by contrast, they are inhibited by both the SIVmac and SIVagmVif proteins [90]. However, human A3G (hA3G) and chimpanzee A3G (CPZ A3G) are inhibited by the HIV-1 Vif protein but are resistant to Vif of SIVagm [90]. This species-specific activity of Vif likely limits transmission of lentiviruses to humans, apes, and other nonhuman primates. Interestingly, SIVcpz and SIVsm can overcome human A3G antiviral activity [8], suggesting a possible role for Vif mediated counteraction of A3G in cross-species transmission of SIVcpz and SIVsm into the human population and emergence of HIV-1 and HIV-2. In 2004, four independent groups showed that the amino acid at position 128 in A3G (D in hA3G and K in AGM A3G) confers species specificity [91,92,93,94]. Mutation of D to K in hA3G rendered it sensitive to inhibition by the SIV Vif protein and resistant to HIV-1 Vif. On the other hand, changing K to D in AGM A3G made it sensitive to the HIV-1 Vif protein but resistant to the SIV Vif protein [91,92,93,94]. Amino acids 14 to 17 (DRMR) in the HIV-1 Vif and SERQ or SEMQ found in SIVagmVif likely play a role in mediating the interaction with the amino acid at position 128 in both hA3G and AGM A3G, respectively. Substituting DRMR in the HIV-1 Vif with SERQ or SEMQ, enables it to degrade both the AGM and RM A3G proteins but abolishes the interaction with hA3G. Similarly, changing the SERQ residues to DRMR in SIVagmVif redirects targeted degradation from AGM A3G to hA3G. The species-specific activity of HIV-1 Vif is likely governed by the interaction of positive residues in DRMR with the negatively charged D128 in hA3G [95]. Overall, these studies support a hypothesis that Vif-mediated inhibition of macaque APOBEC3 family proteins could be important for cross-species transmission of HIV-1 to macaques. 

### 4.3. TRIM5α and TRIMcyp

HIV-1 does not infect Old World macaques such as RM and CMs due to a post-entry block that occurs before reverse transcription [96,97]. This post-entry block was initially named lentivirus restriction factor 1 or Lv1. AGM cells also exhibit a similar block to lentiviruses, including HIV-1, HIV-2, EIAV, and SIVmac [98,99]. In 2004, Stremlau *et al*. identified TRIM5α as the inhibitory factor by screening a rhesus macaque complementary DNA (cDNA) expression library for genes that would block HIV-1 infection in human cells [100]. TRIM5α belongs to the tripartite motif (TRIM) family of proteins that contain: (i) a RING finger, which presumably has E3 ubiquitin ligase activity; (ii) a B-box2 domain, which plays a role in higher order self-association of TRIM5α oligomers; and (iii) a coiled-coil domain, which is required for dimerization of TRIM5α. Interestingly, TRIM5α also contains a C-terminal B30. 2/SPRY domain that plays a role in the detection of incoming viral capsid proteins [101]. Following recognition of the viral capsid, TRIM5α links the viral core to an ubiquitin-proteasome-dependent pathway. This results in disruption of the preintegration complex and suppression of reverse transcription [101]. Several reports also suggest a proteasome independent pathway of HIV-1 restriction by TRIM5α. If the proteasome pathway is inhibited, HIV-1 infection proceeds through reverse transcription; however, nuclear entry of viral DNA is impaired [102,103]. 

New World monkey cells, such as those of Owl monkeys (OWM, *Aotus trivirgatus*), also exhibit a post-entry barrier to HIV-1 infection [104]. Interestingly, a novel TRIM5-cyclophilin A fusion protein (TRIMcyp) was identified as the inhibitory molecule preventing HIV-1 infection in this species [105,106]. The TRIMcyp fusion protein arose with LINE-1 mediated retrotransposition of the cyclophilinA cDNA into the TRIM5 locus [105]. The cyclophilinA domain in the TRIMcyp protein functions like the B30. 2 domain of TRIM5α and binds to the incoming HIV-1 capsid. Interestingly, cyclophilin A, which interacts with the proline rich loop on the surface of the HIV-1 capsid, has been shown to enhance HIV-1 replication in human cells [107,108]. However, cyclophilin A in Old World monkey cells blocks HIV-1 infection. Two reports suggest that cyclophilinA renders HIV-1 sensitive to the antiviral action of TRIM5α in Old World macaques, although the mechanism of action is not entirely clear [109,110]. 

Both TRIM5α and TRIMcyp proteins exhibit species-specific restriction. This species specificity is determined by the sequence variations in the B30. 2/SPRY or cyclophilinA domain in different monkey species and amino acid differences in the viral capsid [111,112,113]. Both RM TRIM5α and CM TRIM5α restrict HIV-1 but not SIVmac; whereas human TRIM5α is less effective against HIV-1 and SIVmac, but potently restricts N-tropic murine leukemia virus (N-MLV) [100,111,114]. AGM TRIM5α shows restriction activity against HIV-1, HIV-2, and SIVmac, but it fails to inhibit SIVagm [111,115,116]. In the case of TRIMcyp proteins, OWM TRIMcyp interferes with HIV-1 but not SIVmac [105,106], while the RM TRIMcyp restricts HIV-2 and SIVagm but does not inhibit either HIV-1 or SIVmac [112,117]. Interestingly, neither the PTM nor the CM TRIMcyp blocks either HIV-1 or SIVmac239 infection [118]. However, PTM TRIMcyp restricts FIV, HIV-2 and SIVagm infection [112]. These studies suggest that both the TRIM5α and TRIMcyp proteins may also contribute to controlling cross-species transmission of lentiviruses. 

### 4.4. BST2/Tetherin/CD317

Bone marrow stromal antigen 2 (BST2), also known as tetherin, CD317, or HM1.24 antigen, is a type II membrane protein with an amino-terminal cytoplasmic tail, a single transmembrane domain, an extracellular coiled-coil domain, and a GPI membrane anchor at the C-terminus [119]. In 2008, BST2 was identified as an interferon (IFN) inducible, Vpu sensitive factor that could interfere with the release of HIV-1 progeny virions from the surface of cells [120,121]. In cell types that constitutively express BST2, such as primary CD4^+^ T-cells, HeLa, and Jurkat cells, Vpu is required for the efficient release of progeny virions from the cell surface. However, Vpu is not required for release of progeny virions in cell types such as 293T and HT1080 cells which do not express BST2 [120,121]. BST2 retains fully formed viruses to the surface of cells by linking them to the cell membrane and to each other [120]. These cell surface attached virions are re-internalized via recycling of BST2. They then traffick to late endosomes/lysosomes for degradation [121,122,123]. Whether BST2 decreases cell-to-cell transmission of HIV-1 is unclear because it has been shown to either reduce cell-to-cell transmission [124,125] or to be unable to inhibit cell-to-cell spread of virus [126,127]. 

Lentiviruses have evolved different strategies to overcome BST2-mediated restriction. The known antagonists of BST2 include the Vpu protein of HIV-1, envelope (Env) protein of HIV-2 and SIV, and the Nef protein of SIV. HIV-1 Vpu has been shown to reduce the levels of BST2 expression on the cell surface [121,123]. However, the mechanism of downregulation is not entirely clear. Recent reports suggest that Vpu, which is also an integral membrane protein like BST2, targets the TM domain of BST2. However, the site at which Vpu interacts with BST2 is not very clear. Vpu has also been shown to reduce total cellular levels of endogenous as well as exogenously expressed BST2. Several reports suggest involvement of either a beta-TrCP dependent proteasome or endolysosomal pathway in Vpu-mediated degradation of BST2 [128,129,130,131,132]. In contrast to HIV-1 Vpu, HIV-2 Env and SIV Nef interact with the BST2 ectodomain [133] and cytoplasmic tail [134,135], respectively. Furthermore, they may induce downregulation of BST2 from the cell surface without causing degradation [133,134,135]. 

HIV-1 Vpu exhibits species-specific activity against BST2. It can overcome the activity of human and chimpanzee BST2, but is ineffective against BST2 from RM, AGM, and mustached monkeys (*Cercopithecus cephus*) [59,132,134,136,137,138]. Vpu expressed from SIVmus, which infects mustached monkeys, also exhibits species-specific function. It is effective in antagonizing mustached monkey and AGM BST2, but is not active against human BST2 [7,139]. Surprisingly, the Vpu proteins expressed by SIVcpz and SIVrcm lack activity against all BST2 proteins tested [7,139]. Interestingly, the Nef proteins from these viruses downregulate BST2 expression [7]. The SIV Nef also exhibits species-specific activity against BST2. It targets RM, PTM, and AGM BST2 but cannot antagonize human BST2 [134,135,139]. This is due to the absence of a G/DDIWK motif in human BST2, which the SIV Nef targets in the RM protein [134,135]. 

The importance of counteracting BST2 for persistent viral replication is highlighted by the fact that multiple lentiviral proteins have evolved to antagonize BST2. However, the requirement for Vpu-mediated antagonism of BST2 for HIV-1 replication and pathogenesis still requires additional investigation. This is because Vpu may not be absolutely required for replication. For example, there are primary isolates of HIV-1 that do not express Vpu due to a mutation in its start codon [140,141]. Furthermore, although Vpu and Nef proteins of Group O HIV-1 are poor BST2 antagonists, the viruses still cause AIDS in infected individuals [7]. Vpu has also been shown to enhance virion release even in the absence of cell surface down modulation and intracellular depletion of BST2 [142]. Finally, as indicated previously, there are also observations showing that the absence of Vpu reduces virion release from cells but does not affect cell-to-cell transmission of the virus, which is suggested to be a major mode of HIV-1 transmission *in vivo*. 

### 4.5. SAMHD1

Myeloid lineage cells such as monocytes, dendritic cells and macrophages are known to be refractory to HIV-1 infection [143,144,145,146]. However, infection of these cells with HIV-1 can be enhanced by preincubating the cells with virus-like particles derived from SIV that express the Vpx protein from either the HIV-2 or SIVsm lineage [144,147]. *Vpx*, which likely evolved as a result of duplication of the *vpr* gene [148], has been shown to interact with the DDB1-CUL4-DCAF1 E3 ubiquitin ligase complex and enhance HIV-1 infection of DCs, monocytes, and macrophages by causing proteasomal degradation of a putative restriction factor [144,149,150]. Recently, two independent groups identified sterile alpha motif (SAM) and histidine/aspartic acid (HD) domain containing protein 1 (SAMHD1) as a novel restriction factor that inhibits HIV-1 infection of myeloid cells. They showed that Vpx interacts with SAMHD1, resulting in its degradation via the proteasome [151,152]. Although the biological function of SAMHD1 is not very clear, mutations in SAMHD1 have been associated with Aicardi-Goutieres syndrome (AGS), a genetic encephalopathy with symptoms mimicking viral infection. This disease is characterized by excessive production of IFNα and chronic lymphocytosis [153]. Interestingly, SAMHD1 is induced by IFN and has been proposed to act as a negative regulator of the IFN response [153]. Mutations in other genes such as three prime repair exonuclease 1(TREX1), and components of the endonuclease complex RNase H2 (RNAseH2A, RNAseH2B, and RNAseH2C), have also been linked to AGS [154,155]. However, in contrast to SAMHD1, TREX1 and RNAseH2A have been shown to facilitate HIV-1 infection [156,157]. Since, TREX1 and RNaseH2 complex are involved in nucleic acid metabolism, a similar role for SAMHD1 was suggested. Indeed, two recent reports suggest that the dimeric SAMHD1 core domain has 2’-deoxy-guanosine 5’-triphosphate (dGTP)-stimulated deoxynucleoside triphosphate (dNTP) triphopsphohydrolase activity [158,159]. They showed that SAMHD1 converts dNTPs to deoxynucleotides and triphosphate in an *in vitro* enzymatic assay. Interestingly, Vpx expression or SAMHD1 depletion increases the amount of dNTPs in macrophages, suggesting that SAMHD1 inhibits HIV-1 infection by decreasing the dNTP pool required for viral cDNA synthesis [160]. 

Recent reports also suggest that antagonism of SAMHD1 by Vpx occurs in a species-specific manner [61,62]. Human and gibbon SAMHD1 can be degraded by Vpx proteins from HIV-2rod, SIVmac, and SIVsm but not by Vpx from SIVrcm and SIVmnd2. However, all these Vpx proteins can induce degradation of rhesus macaque and mangabey SAMHD1 [62]. Interestingly, none of these Vpx proteins induce degradation of SAMHD1 from gray mouse lemurs, which is the most distantly related primate from humans [62]. Surprisingly, some of the lentiviral Vpr proteins also degrade SAMHD1 [61]. Vpr protein from SIVdeb, which infects De Brazza’s monkeys, can not only degrade SAMHD1 from De Brazza’s monkeys but also from human, SM, AGM, RM, RCM, and mandrills [61]. Similarly, Vpr from SIVmus can also degrade SAMHD1 from diverse primate species [61]. Interestingly, Vpr from SIVagm Grivet (677 strain) only degrades SAMHD1 from AGMs, whereas SIVagmVervet(9648 strain) Vpr is able to degrade SAMHD1 from AGM, RM, RCM, SM, De Brazza’s monkeys, and Mandrills [61]. Overall, these results suggest that primate lentiviruses have evolved strategies to overcome SAMHD1 restriction. 

Interestingly, HIV-1 does not have a mechanism to antagonize human SAMHD1, but it still replicates and causes disease in humans. This raises questions about the importance of SAMHD1 mediated restriction of HIV-1 in myeloid cells. Since mutations in SAMHD1 result in increased production of interferon, it can be hypothesized that SAMHD1 mediated restriction of HIV-1 infection in myeloid cells likely prevents an innate immune response. Indeed, forcing HIV-1 to replicate in DCs by providing Vpx *in trans* results in interferon production [161], suggesting that HIV-1 may not have evolved a mechanism to inhibit SAMHD1 in order to avoid productively infecting myeloid cells and inducing an immune response. However, SIVmac, which causes disease in RMs, expresses Vpx to disrupt RM SAMHD1 restriction activity. Furthermore, new data indicate that Vpx-mediated degradation of SAMHD1 is necessary for macrophage-tropism and robust replication of SIVmne in CD4+ T-cells in PTMs [29]. Therefore, it is not clear why HIV-1 has not evolved a mechanism to counteract SAMHD1 and enhance infection of myeloid cells. Further studies will be necessary to understand the importance of myeloid cell infection for persistent HIV-1 and SIV replication. 

### 4.6. Role of Interferon

Endocytosis of HIV-1 induces IFNα in plasmacytoid dendritic cells via TLR7 recognition of genomic viral RNA [162]. Recently, Manel *et al.* suggested that an intrinsic HIV-1 recognition mechanism in monocyte-derived dendritic cells plays a role in detection of newly synthesized viral capsid proteins, thereby activating the innate immune response [161]. Interestingly, macrophages may lack expression of a pattern recognition receptor for HIV-1, as these cells can be infected with HIV-1 without triggering an innate immune response [163]. Induction of IFNα results in the expression of many interferon stimulated genes (ISGs) and the establishment of an antiviral state in the cell. Significantly, retroviral restriction factors (*i.e.*, APOBEC3 family proteins, TRIM5α, BST2, and SAMHD1) are upregulated by type I interferons [122,153,164,165,166]. Several studies have also identified additional ISGs that inhibit HIV-1 infection. For example, TRIM22 has been shown to inhibit HIV-1 transcription [167,168], and may disrupt Gag assembly [169]. Interferon inducible transmembrane (IFITM) proteins inhibit HIV-1 infection either by suppressing Gag production (IFITM1) or by interfering with virus entry (IFITM2 and IFITM3) [170]. Type I interferons have also been shown to increase expression of TRIM5α and TRIMcyp in monkey cells. Increased expression of TRIMcyp in OWM cells was also shown to restrict HIV-1 infection [166]. Additionally, early reports suggested that pretreatment of cells with type I interferons inhibits the replication of HIV-1 by interfering with reverse transcription and integration of the provirus [171,172,173,174,175,176,177]. Thus, it is likely that the upregulation of retroviral restriction factors mediates inhibition of HIV-1 replication by interferon. It therefore can be hypothesized that interferon induced restriction factors have forced the lentiviruses to evolve strategies to overcome the inhibitory activities of ISGs in order to infect and persistently replicate in the host. 

## 5. Macaque-Tropic HIV-1

### 5.1. Rational Design of Macaque Tropic-HIV-1

The identification of retroviral restriction factors and the counter measures used by primate lentiviruses have guided the rational design of macaque-tropic HIV-1 (mtHIV-1) clones (Table 2). HIV-1 can replicate in human cells because its capsid is not recognized by the restriction factor human TRIM5α, and its Vif protein inhibits APOBEC3 protein family members. However, as discussed above, the RM TRIM5α protein blocks HIV-1 infection, and the Vif protein cannot counteract the RM APOBEC3 family of restriction factors. These data suggest that the inability of HIV-1 to replicate in RM cells may be overcome by engineering resistance to TRIM5α and APOBEC3 proteins. Thus, a mtHIV-1 virus was initially developed by substituting the HIV-1 *capsid* and *vif* sequences with the corresponding sequences from SIVmac [178]. mtHIV-1, which has 90% of the HIV-1 genome combined with 10% of SIV sequences, replicated robustly in a RM T-cell line and RM PBMCs after *in vitro* adaptation [178]. However, the replication efficiency of mtHIV-1 *in vivo* is unknown. To minimize the sequences from SIV, an HIV-1 derivative which carries only the SIVmac*vif* gene and a short 21 base pair segment from the SIV capsid sequence corresponding to the HIV-1 cylophilin A binding loop was engineered and showed increased infectivity in a CM T-cell line [179]. Only after passaging this virus in the CM T-cell line was an *in vitro* adapted HIV-1 derivative (NL-DT5R) generated that could replicate well in the CM T-cell line as well as CD8^+^ T-cells depleted T-cells from both PTMs and RMs. Although NL-DT5R established a productive infection and elicited humoral responses in PTMs, it did not cause CD4^+^ T-cells depletion or disease [180]. A modified version of this virus with enhanced replication capacity in CM CD4+ T-cells was also generated by splicing a loop between alpha helices 6 and 7 (L6/7) of the SIVmac CA into the corresponding region in HIV-1(NL-DT5R6/7S) [181]. Interestingly, although NL-DT5R also established infection in CMs, viral loads were marginal and disappeared by 4 weeks post-infection [182]. Long-term passaging of both CXCR4- and CCR5-tropic NL-DT5R in CM-derived HSC-F cells resulted in a total of 14 mutations. These mutations were introduced into the parental NL-DT5R clone to generate a clone named MN4-5. A modified MN4-5 clone (MN4-5S) generated by inserting the loop between alpha helices 6 and 7 (L6/7) of the SIVmac CA into the corresponding region in HIV-1 showed enhanced replication compared to the parental NL-DT5R and MN4-5 clones in CM-derived HSC-F cells and CD8^+^T-cells depleted PBMCs from CMs. Although infection of CMs with MN4-5S resulted in higher viral titer compared to NL-DT5R at 2 to 3 weeks post infection, viremia became undetectable at 6 weeks post infection, partly due to control by CD8^+^T-cells [182]. 

### 5.2. Unique Susceptibility of Pigtailed Macaques to HIV-1 Infection

PTMs are known to be uniquely susceptible to HIV-1 infection [183,184,185,186,187]. Agy *et al*. first showed that PTMs can be infected with HIV-1 [183]. All the infected animals demonstrated seropositivity against a broad range of HIV-1 proteins. Furthermore, the authors were able to detect viral DNA in PBMCs and were able to re-isolate virus by cocultivation. However, viral loads diminished rapidly after inoculation. In another study, Gartner *et al*. infected PTMs with autologous cells expressing low amounts HIV-1 [185]. During the first 10 weeks post infection, they were able to recover infectious virus from PBMCs and lymph nodes in 3 out of 4 infected macaques. In one of the infected animals, virus could be re-isolated at 38 and 61 weeks post-infection, suggesting that the animal was persistently infected with HIV-1. Interestingly, *in vivo* passaging of the virus in PTMs did not select for pathogenic variants. This is probably because PTMs restrict HIV-1 replication to a level that is not sufficient for adaptive mutations to occur. Although HIV-1 replicates more vigorously in newborns, passaging of HIV-1 in newborn PTMs did not result in the emergence of pathogenic variants [188,189]. Despite these outcomes, these studies demonstrated that PTMs are susceptible to HIV-1 infection. We have also observed that PTM PBMCs can be more easily transduced with VSVG pseudotyped HIV-1 than RM PBMCs, suggesting the absence of a post-entry block [190]. Recently, several groups, including ours, have observed that this unique susceptibility of PTMs to HIV-1 infection is due to the absence of the retroviral restriction factor, TRIM5α. Instead PTMs express novel isoforms of TRIM5 (TRIM5θ, which lacks the B30. 2(SPRY) domain and TRIM5η, which has a deletion of the entire exon 7) that do not restrict HIV-1 infection [191]. Furthermore, PTMs express a TRIM5-cyclophilin A fusion protein (TRIMcyp), which resulted from a LINE-mediated retrotransposition of the cyclophilin A cDNA into the untranslated region of exon 8 of the TRIM5 locus [112,118,192,193]. The PTM TRIMcyp protein is similar to the OWM TRIMcyp. However, it does not restrict HIV-1 infection, although it inhibits FIV, HIV-2 and SIVagm [112,118,192,193]. A comparison of the antiviral action of OWM TRIMcyp and PTM TRIMcyp showed that a single amino acid mutation (H69R) in the cyclophilinA domain of PTM TRIMcyp prevents it from interacting with the HIV-1 CA [112]. 

**Table 2:** Macaque tropic HIV-1 derivatives.

Clone Name (Backbone)	Substitutions Made	Passaging of Viruses	*In vitro* Replication	*In vivo* Replication	Disease	References
stHIV-1 (NL4-3)	SIVmac239 capsid and *vif*	Yes	Replicated robustly in RM T-cells (221) after *in vitro* passaging	?	?	[178]
NL-DT5R (NL4-3)	Cyclophilin A binding domain of SIV capsid and *vif*	Yes	Shows spreading infections in CM T-cell line and CD8^+^ T-cells depleted T-cells PTMs	Viremia becomes undetectable within 11 and 4 weeks post infection in PTMs and CMs respectively	No	[179,180]
NL-DT5R6/7S (NL4-3)	Modified version of NL-DT5R	No	Replicates in CD8^+^T-cells depleted T-cells from CMs	?	?	[181]
MN4-5S (NL4-3)	Modified version of NL-DT5R	No	Replicates in CD8^+^T-cells depleted PBMCs from CMs	Viremia becomes undetectable after 6 weeks post-infection in CMs	?	[182]
stHIV-1_sv_, stHIV-1_2v_ (NL4-3)	SIVmac *vif* or HIV-2 *vif* and SHIV_KB9 _*env*	No	Shows spreading infections in PTM PBMCs	Viremia becomes undetectable after 25 weeks post-infection in PTMs	No	[194]
HSIV-vif (NL4-3)	SIVmne027 *vif*	No	Replicates efficiently in PTM PBMCs	Viremia detectable through 2 years post-infection in PTMs	No	[190]
HSIV-vif-Yu2 (BRU-YU2)	SIVmne027 *vif*	No	Replicates efficiently in PTM PBMCs	?	?	[190]
HSIV-vif-AD8 (NL-AD8)	SIVmne027 *vif*	No	Replicates efficiently in PTM PBMCs	?	?	[190]

### 5.3. HIV-1 Derivatives with a Minimal SIV vif Substitution

Absence of a post-entry block suggested that inhibition of HIV-1 replication in PTM CD4^+^ T-cells may be mainly due to APOBEC3 family proteins. This presents an advantage for the development of a minimally modified HIV-1 that can potentially infect and cause AIDS in a macaque species. Since SIVmac Vif can degrade RM APOBEC3G, Hatziioannou *et al*. constructed an HIV-1 derivative carrying SIVmac *vif* in place of HIV-1 *vif* [194]. They also constructed an HIV-1 derivative with an HIV-2 *vif* gene substitution. Inoculation of PTMs with an admixture of these two viruses resulted in acute infection and viremia persisted for 25 weeks post infection. However, infection was controlled thereafter and it did not result in CD4^+^T-cell depletion. In a separate experiment, we also constructed an HIV-1 derviative carrying the *vif* gene from SIV (HSIV-vif) [190], except we used a *vif* gene derived from the highly pathogenic PTM-adapted SIVmne027 [195,196]. Compared to the previous studies, our study showed extended viral replication through 44 weeks post infection and small rebounds in viral titer at 64 and 72 weeks post-infection in juvenile PTMs. Furthermore, viral DNA could be detected in PBMCs through 90 weeks post-infection, suggesting that the animals were persistently infected for nearly 2 years. Infection of newborn PTMs also resulted in viremia that was detectable through at least 40 weeks post-infection. Finally, we have continued to monitor these animals for more than 3 years. We have observed very low plasma viral loads. Surprisingly, one animal has declining CD4^+^counts, suggesting disease progression. Interestingly, for reasons that are unclear, newborn PTMs infected as newborns appear to control their infection earlier than those animals infected as juveniles [197].

The reasons for the extended period of replication of HSIV-vif in PTMs compared to the other mtHIV-1 clones is unknown, but could be due to differences in the construction of the viruses. The viruses made by Hatziioannou *et al*. contained an *env* gene from RM adapted SHIV_KB9_ and *vif* gene from SIVmac239 or HIV-2_ROD_. Although we used the same back bone, HIV-1 NL4-3, our clone contained a *vif* allele from PTM adapted SIVmne027 and the NL4-3 envelope. Interestingly, SIVmac239 Vif does not degrade RM A3F [198]. By contrast, we found that SIVmne027 reduces both PTM A3G and A3F in cells and virions [190]. Potentially, this ability of SIVmne027 Vif to effectively degrade both PTM A3G and A3F may have contributed to longer persistent infection in the PTM host. Finally, these studies suggest that mtHIV-1 based on NL4-3 may not be very fit for replication in PTM host. Viral clones based on other HIV-1 variants should be considered in future experiments. 

## 6. Potential Problems with Simian Tropic HIV-1 Derivatives

PTMs infected with different macaque-tropic HIV-1 viruses have not yet developed AIDS, which suggests that there could be other unknown barriers to HIV-1 replication in PTMs. Alternatively, there could be problems associated with the insertion of the SIV *vif* gene in place of HIV-1 *vif* gene. Our results suggested that SIV Vif expression from HSIV-vif may be suboptimal for high-level replication in the PTM host. Indeed, we observed that HSIV-vif did not degrade either PTM A3G or A3F as effectively as pathogenic SIVmne027. As a result, HSIV-vif virions incorporated PTM A3G and A3F at levels higher than those found in wild type SIVmne027 virions [190]. Inefficient degradation of A3G and A3F by HSIV-vif could potentially be a problem in the host where A3G levels are upregulated by the host immune response. Several reports suggest that cytokines, chemokines, and cellular activation events can upregulate both A3G and A3F expression [199,200,201,202,203,204,205,206]. It is possible that Vif expression from HSIV-vif may not be sufficient to cope with changes in A3G/A3F expression and hence increased levels of A3G/A3F could reduce the infectivity of progeny virions. Insufficient inhibition of A3G could also result in greater control of HSIV-vif by the cellular immune response as reported by Casartelli *et al*. [74]. Surprisingly, we did not observe hypermutation in the proviral DNA isolated from PBMCs from infected macaques. However, this could be due to low level of replication of the virus in the host or clearance of hypermutated virus. Finally, our unpublished results suggest that G to A mutated genomes can be isolated from PTM CD4^+^ T-cells infected with HSIV-vif *in vitro*, suggesting that the level of Vif expression may not be sufficient to completely overcome PTM A3 family proteins [197]. 

The absence of virion associated Vif protein is another characteristic distinguishing HSIV-vif from SIVmne027 [190]. This could be because SIV Vif does not incorporate into HIV-1 core particles, or it may be due to a low level of Vif expression from HSIV-vif. Although it is not clear if Vif incorporation into virions is indeed required for pathogenesis of HIV-1 and SIV *in vivo*, we cannot rule out the importance of SIV Vif incorporation into virions in the pathogenesis of HSIV-vif. Several reports suggest that Vif packaging into virions may not be required for APOBEC3 degradation or viral infectivity in cell culture [207,208,209,210,211,212]. However, other reports suggest Vif packaging could be necessary for functions such as G2 cell cycle arrest, modulation of reverse transcription, or inhibition of IRF3 [213,214,215,216]. 

Interestingly, passaging of mtHIV-1 viruses *in vitro* did not generate pathogenic variants. Even after *in vitro* passage, the mtHIV-1 virus generated by Kamada *et al*., showed limited replication capacity in the PTM host [180]. Additional attempts to adapt this virus (NL-DT5R) to CMs did not enhance replication in this alternate species [182], which could be because this virus had poor replication capacity to begin with. All these studies clearly demonstrate that even though these viruses were engineered to overcome specific innate restriction factors, they did not result in high levels of replication in the host. Additionally, the observations that CD8^+^ T-cell depletion caused rebounds in plasma viral loads and that neutralizing antibodies were generated against stHIV-1, support a hypothesis that these viruses may not overcome adaptive immunity in the host [194]. However, in the Igarashi *et al*. study, CD8^+^ T-cell depletion did not enhance viral loads in PTMs [180]. Finally, since *in vitro* passaging did not result in pathogenic variants, we envision that passaging of HSIV-vif *in vivo* might select for escape variants that would avoid recognition by cytotoxic T-cells and neutralizing antibodies. 

An alternative hypothesis to the inability of these viruses to escape from adaptive immune responses is that they may still be susceptible to restriction by innate immune factors. The rationale behind the prototype mtHIV-1 clones addressed escape from TRIM5α and A3 proteins, but not other possible restriction factors. For example, none of the viruses have included a determinant that allows the viruses to evade the inhibitory effects of BST2. Identification of BST2 as a species-specific retroviral restriction factor also suggests that additional modifications to mtHIV-1 derivatives, such as insertion of the SIV *nef* gene in place of the HIV-1 *nef*, may promote its replication in the PTM host. On the other hand, substitution of the HIV-1 *nef* with SIV *nef* may not be necessary because SHIV chimeric viruses with HIV-1 *nef* genes are still pathogenic [217,218,219]. Another possibility is that although Vpx is not required for HIV-1 infection of humans, it may be required for infection of macaques. Indeed, Vpx deletion mutants of SIVs as well as mutants that fail to inhibit SAMHD1 are attenuated in macaques compared to wild type SIVs, suggesting the importance of Vpx in pathogenesis [26,29,30]. 

Viral infections result in interferon induction but it is unknown how mtHIV-1 derivatives respond to interferon induction *in vivo*. We have observed that IFNα potently inhibits replication of HSIV-vif based on NL4-3 in PTM CD4^+^ T-cells, while it has little effect on replication of pathogenic SIVmne027 [220,221]. Thus, it is possible that HSIV-vif does not effectively overcome IFN induced restrictions in PTM CD4^+^ T-cells in the host, resulting in control of viral replication. It will be important to determine whether HSIV-vif or other mtHIV-1 can evolve resistance to IFN-induced restriction and to define the cellular factors mediating inhibition. 

## 7. Concluding Remarks

Significant progress has been made toward understanding the innate barriers that influence cross-species transmission of lentiviruses. Although HIV-1 derivatives that can infect macaques have been developed, none has yet caused disease. However, these chimeras have helped us define key blocks to transmission and persistent infection. Low level of replication of macaque-tropic HIV-1 chimeras *in vivo* suggests that there are likely additional restrictions to replication that remain to be identified. Future studies should address the lack of correlation between viral replication *in vitro* in macaque T-cells and *in vivo*. An in depth understanding of the factors that control replication of macaque-tropic HIV-1 clones *in vivo* should provide insights into the role of the innate immune response in cross-species transmission and further progress toward a nonhuman primate model of HIV-1 infection and disease. 
